# Speech Discrimination Tasks: A Sensitive Sensory and Cognitive Measure in Early and Mild Multiple Sclerosis

**DOI:** 10.3389/fnins.2020.604991

**Published:** 2020-12-23

**Authors:** Pippa Iva, Joanne Fielding, Meaghan Clough, Owen White, Branislava Godic, Russell Martin, Ramesh Rajan

**Affiliations:** ^1^Department of Physiology, Biomedicine Discovery Institute, Monash University, Melbourne, VIC, Australia; ^2^Department of Neuroscience, Central Clinical School, Monash University, Alfred Centre, Melbourne, VIC, Australia

**Keywords:** central auditory processing, auditory attention, multiple sclerosis, sensory impairment, cognitive impairment, early disease biomarker, speech in noise perception

## Abstract

There is a need for reliable and objective measures of early and mild symptomology in multiple sclerosis (MS), as deficits can be subtle and difficult to quantify objectively in patients without overt physical deficits. We hypothesized that a speech-in-noise (SiN) task would be sensitive to demyelinating effects on precise neural timing and diffuse higher-level networks required for speech intelligibility, and therefore be a useful tool for monitoring sensory and cognitive changes in early MS. The objective of this study was to develop a SiN task for clinical use that sensitively monitors disease activity in early (<5 years) and late (>10 years) stages of MS subjects with mild severity [Expanded Disability Status Scale (EDSS) score < 3]. Pre-recorded Bamford-Kowal-Bench sentences and isolated keywords were presented at five signal-to-noise ratios (SNR) in one of two background noises: speech-weighted noise and eight-talker babble. All speech and noise were presented via headphones to controls (*n* = 38), early MS (*n* = 23), and late MS (*n* = 12) who were required to verbally repeat the target speech. MS subjects also completed extensive neuropsychological testing which included: Paced Auditory Serial Addition Test, Digit Span Test, and California Verbal Learning Test. Despite normal hearing thresholds, subjects with early and late mild MS displayed speech discrimination deficits when sentences and words were presented in babble – but not speech-weighted noise. Significant correlations between SiN performance and standardized neuropsychological assessments indicated that MS subjects with lower functional scores also had poorer speech discrimination. Furthermore, a quick 5-min task with words and keywords presented in multi-talker babble at an SNR of −1 dB was 82% accurate in discriminating mildly impaired MS individuals (median EDSS = 0) from healthy controls. Quantifying functional deficits in mild MS will help clinicians to maximize the opportunities to preserve neurological reserve in patients with appropriate therapeutic management, particularly in the earliest stages. Given that physical assessments are not informative in this fully ambulatory cohort, a quick 5-min task with words and keywords presented in multi-talker babble at a single SNR could serve as a complementary test for clinical use due to its ease of use and speed.

## Introduction

Multiple sclerosis (MS), a debilitating disease of the central nervous system (CNS), is the most common cause of neurological disability in young adults (Wallin et al., 2019). People with MS (pwMS) display a range of motor, sensory and cognitive symptoms that can sometimes cause serious disability, although occasionally can be mild (Huang et al., 2017; Fielding and Clough, 2019). Currently, the gold standard clinical measure of MS disability is the Expanded Disability Status Scale (EDSS) (Kurtzke, 1983). While the EDSS provides a sound measure of motor dysfunction, particularly later in the disease when symptoms are more pronounced, it has been less reliable at detecting early symptomology (EDSS scores of <3 – early disease, low disability), which are often subtle and difficult to quantify objectively (Amato and Ponziani, 1999; Cinar and Yorgun, 2018). In particular, the EDSS does not characterize cognitive impairment, which is a particularly debilitating component of the disease, affecting 40–70% of pwMS (Chiaravalloti and DeLuca, 2008) manifesting at all disease stages, including onset (Brissart et al., 2013; Ruet et al., 2013) and often predating physical symptoms (McNicholas et al., 2017). Consequently, there is a need for reliable and objective measures of early MS that are sensitive to the range of symptoms that may occur, particularly for clinical management as evidence suggests that insidious progression during the early phase of MS can meaningfully inform prognostication (Lublin et al., 2003; LoPresti, 2018). Furthermore, sensitive measures of disease surveillance also provide a means to evaluate treatment effects of potential and current therapeutics. We propose an innovative approach using speech-in-noise (SiN) assessments to monitor early disease activity. SiN is a complex process that integrates sensory information and cognitive processing, that maybe be measured with high sensitivity, allowing a comprehensive measure of sensory and cognitive function in early MS.

Speech is a complex sound consisting of rapidly changing elements that require precise temporal detection within milliseconds for identifying consonants or voice onset time, especially in the presence of background noise (Anderson et al., 2010). Early stages of SiN processing take place subcortically within the auditory brainstem, where both monoaural and binaural (two ears) sensory information processing forms a necessary element of segregating an ambiguous sound mixture into coherent auditory objects (Anderson and Kraus, 2010). Early brainstem involvement is common in MS and accounts for 20% of symptoms supportive of a diagnosis of Clinically Isolated Syndrome (CIS) (Habek, 2013), the earliest stage of disease in 85% of patients who subsequently develop MS (Miller et al., 2005). In MS, demyelination in the brainstem causes slowed conduction velocity and neural dyssynchrony, resulting in less precision to detect acoustic timing cues within the millisecond range (Rappaport et al., 1994; Matas et al., 2010). Background noise simultaneously presented with speech further degrades neural synchrony by disrupting the representation of temporal characteristics of the stimulus (Anderson et al., 2010; White-Schwoch et al., 2015), potentially making SiN assessments a potent measure for detecting MS changes. It’s possible that such MS-induced deficits in processing timing cues create significantly more degradation of the target speech compared to healthy controls.

Degraded signals in adverse listening environments force the listener to engage a range of cognitive processes for top–down strengthening of the target signal (Stenfelt and Ronnberg, 2009; Zekveld et al., 2013). A cognitive process of great relevance in SiN processing is attention, in particular, the conscious and active attentional processes that have a robust top-down effect on most of the auditory pathway processes (Vanthornhout et al., 2019). In the context of the ‘cocktail party’ phenomenon described by Cherry (1953), attention involves an interplay of bottom–up salience and top–down attention: a listener can ignore other speakers in favor of a target speaker, but when salient information arises, such as the listener’s name, attention switches to the new speaker involuntarily (Cherry, 1953). Attentional systems can be divided into three interconnected subsystems: (a) *alerting*, (b) *orientating*, and (c) *executive* (Posner and Petersen, 1990). In the context of SiN processing, *alerting* is driven by bottom-up cues that draw the listener’s attention to the salient acoustic cues of the target speech and requires a state of alertness to prepare attention to an expected signal (Petersen and Posner, 2012). *Orientating* refers to the ability to prioritize speech coming from a specific location in space, and reduce the interference caused by a masker at a different location (Posner and Petersen, 1990; Petersen and Posner, 2012). Spatial cues are particularly useful salient cues for the auditory scene analysis, and several studies have demonstrated that speech recognition in noise improves when the source of the speech is separated horizontally from the interference (Plomp and Mimpen, 1981; Freyman et al., 1999, 2001; Culling et al., 2004; Litovsky, 2012; Yost, 2017). *Executive processes* are important for SiN perception and have been previously described by Dryden et al. (2017) in three subdomains: (a) *set-shifting*, (b) *inhibitory control*, and (c) *updating*, or *working memory*. *Set-shifting*, as the name suggests, is the ability to switch between tasks, or in the context of SiN processing, switch between different target speakers (Miyake et al., 2000). *Inhibitory control* refers to the process of ignoring a distracting interference in order to focus on the desired target (Diamond, 2013). Numerous and simultaneous distracting speech can involuntarily capture the attention of listeners and are thereby likely to place a greater cognitive load on the listener. Complex aspects of attention such as selective, divided, and alternating attention are most often impaired in MS – and even CIS – while the simplest form, attention span, remains generally intact (Amato et al., 2008, 2010). Poor inhibitory control in pwMS may increase susceptibility to distraction by competing noises (Janse, 2012; Dryden et al., 2017). Consequently, the use of multifaceted sensory-and-cognitive SiN processing tasks may offer a sensitive method for capturing attentional deficits in early, minimally disabled MS.

Another prominent cognitive process of great relevance in SiN processing is *working memory*, the limited-capacity temporary storage system for active maintenance of information in the face of ongoing processing and distractions (Ronnberg et al., 2013; Zekveld et al., 2013; Füllgrabe and Rosen, 2016; Dryden et al., 2017; Peelle, 2018). In the cognitive hearing sciences, the ease of language understanding (ELU) model (Ronnberg et al., 2013) emphasizes the subtle balancing act between bottom–up and top–down aspects of language processing and how and when working memory is engaged to support the active maintenance of acoustic information in adverse conditions (Ronnberg et al., 2013). Multifaceted working memory processes integrate lexical and phonological memory stores to ‘fill in the gaps’ of degraded sounds or mismatches between the perceptual speech input and phonological representations stored in long term memory (Ronnberg et al., 2013). Working memory representations are vital for accessing semantic and syntactic relations amongst words and sentences to construct meaningful and coherent speech, and could also serve as templates that guide behavior and bias perceptual activity (Sreenivasan et al., 2011). Working memory impairments are widely reported in early MS (McNicholas et al., 2017), however, working memory is not a unitary construct but a complex of several levels of processing broadly categorized as: maintenance and manipulation. Some studies have reported that pwMS have problems associated with maintenance in working memory, whilst others have concluded that the primary deficit is at the level of the central executive which controls and manipulates the contents of the working memory stores (DeLuca et al., 2004). Further, information processing speed, the speed and efficiency with which information is processed and integrated with other cognitive processes for formation of a behavioral response (Drew et al., 2009; Costa et al., 2017), is prominently slower in early MS and has been proposed to be the underlying factor in cognitive domain deficits such as working memory and attention (DeLuca et al., 2004; Forn et al., 2008). Thereby, slowed information processing speed may in part contribute to a greater cognitive load experienced by MS listeners in SiN conditions compared to healthy controls.

A SiN task comprises the registration of auditory input, the deployment of central cognitive resources to extract the speech of interest (Mattys et al., 2012), and finally the generation of a verbal response that repeats the targeted speech of interest. A cognitive test commonly used in MS is the Paced Auditory Serial Addition Test (PASAT); a complex test of working memory, mental arithmetic, and information speed, that similarly requires the registration of auditory input, the deployment of central cognitive resources, and the generation of a verbal response (Gronwall and Sampson, 1974). Despite its inclusion in the multiple sclerosis functional composite (MSFC), due to its sensitivity to neurocognitive effects of MS (Cutter et al., 1999), there are concerns about whether PASAT deficits are related to the ability to perform the task or to the ability to accurately learn the instruction set for an unfamiliar task (Coo et al., 2005). Many participants use a ‘chunking’ strategy which reduces cognitive demand, casting doubt on the reliability of scores (Fisk and Archibald, 2001; Tombaugh, 2006). There are also persistent complaints that the PASAT is unpleasant and stressful, with one study finding 14.2% of participants were unwilling or failed to complete the test (Coo et al., 2005), and even healthy controls reacting with aversion (Mathias et al., 2004). It is also worth noting that while simpler tasks that present digits aurally, like the Forward and Backward Digit Span Tests, place a lesser load on working memory, these are insensitive to the subtle changes seen in people with early stage MS (Rao, 1986; Landrø, 2000). This highlights the need for a task with significant cognitive load to demonstrate changes in early stage MS, and we propose that a task with high familiarity would provide greater confidence that participants were completing the task as intended.

Here, we evaluated whether early stages of cognitive decline in pwMS (with normal hearing), can be detected using a SiN discrimination task. Our task is ethologically relevant, with high familiarity, and so required little pre-training and was highly relevant to everyday life where we routinely process speech in backgrounds of noise. We propose that our SiN task engages a broader set of cognitive processes and places greater cognitive load than a clear speech task. Using sentences (compared to single words) and modulated noise, requires accessing stored lexical knowledge and integrating it with new, partially degraded information to improve comprehension (Mattys et al., 2009; Zekveld et al., 2013). This relies heavily on working memory (Akeroyd, 2008). Here, we employed complete speech sounds of words or sentences over a wide range of signal-to-noise ratios (SNRs), modeling conditions of high clarity through to near incomprehension. We also employed two different background types; one that causes “energetic masking” to diminish audibility of a target from interference of shared spectro-temporal acoustic signals in the lower levels of the auditory system (Mattys et al., 2009), and an eight-talker babble involving energetic interference but also “informational masking” that produces high-level attention competition effects due to confusability of similar target and masker (Cherry, 1953; Mattys et al., 2012; Kidd and Colburn, 2017). An important factor in the potency of babble as a masker is the number of talkers. Eight competing talkers is the number of talkers confirmed by Simpson and Cooke (2005) to have the most detrimental effect on phoneme detection. The general trend described by these authors was that phoneme detection difficulty increased when the number of competing speakers changed from one to eight, but then decreased after eight and up to 512 speakers (Simpson and Cooke, 2005). With such a large number of speakers, babble noise becomes as similar as temporally flat speech-weighted noise, a purely energetic masker of speech sounds. The distracting effects of numerous onsets and fluctuating amplitude are likely to be sufficiently numerous at eight-talkers and also at their most disruptive, perhaps requiring the listener to devote more attentional resources to monitoring the salient noise (Simpson and Cooke, 2005). As we evaluated early stages of cognitive decline in pwMS, we focused primarily on performance in individuals at the early stages of the disease and only pwMS < 5 years after diagnosis/presentation and EDSS < 3 were evaluated. Individuals with late mild MS (>10 years after onset, EDSS < 3) were also evaluated as physical and cognitive deficits may develop separately over the course of MS (Rahn et al., 2012). Given that cognitive impairments and central auditory processing deficits are reported in early and mildly impaired MS (Migliore et al., 2017), we hypothesized that pwMS would exhibit deficits in the dynamic auditory and cognitive processes underlying SiN discrimination, and that these deficits would predate overt physical disability.

## Materials and Methods

All procedures were approved by the Monash University Human Research Ethics Committee (8170) and conformed to the guidelines of the National Health and Medical Research Council of Australia and the protocols of the Helsinki Declaration for experiments involving human participants.

### Participants

Multiple sclerosis participants were recruited through Royal Melbourne Hospital Australia, and neurologically healthy controls were recruited from the local community. Only patients with relapsing-remitting or CIS were included here; secondary and primary progressive types were excluded. Relapsing-remitting MS patients were defined based on McDonald’s criteria (McDonald et al., 2001) and CIS inclusion was based on the initial neurological disturbance (with varying presentations including visual disturbances, numbness/weakness, and balance problems) and magnetic resonance imaging (MRI) evidence of demyelination. All MS participants were independently mobile, with little to no disability (EDSS of <3) and continued to take all prescribed medication. No patient experienced exacerbated symptomology for at least 3 months prior to participation. Participants were not reimbursed for taking part in the research study; however, they were reimbursed for travel expenses.

Exclusion criteria for both MS and control participants were a history of another neurological disorder, substance abuse/dependence, pregnancy, and/or the presence of hearing loss (see section “Audiometry”). All participants reported English as their native language.

### Neuropsychological Testing

To verify that SiN performance was associated with cognition abilities in MS, neuropsychological testing was conducted in pwMS only. Beck’s Depression Inventory (BDI) (Beck and Steer, 1987), a self-rating inventory of depression, evaluated the presence of depressive symptoms in MS. Total scores between 1 and 10 are considered normal; 11–16 a mild mood disturbance; and any score over 31 suggests severe/extreme depression. The National Adult Reading Test (NART), a test of premorbid intellectual functioning, was used to measure cognitive reserve (Nelson and Willison, 1991). The NART consists of 50 words with atypical phonemic pronunciation, and participants are required to read each aloud (untimed). Higher scores indicate greater cognitive reserve. A modified form of the Fatigue Impact Scale (MFIS) (Fisk et al., 1994) was used for self-reported fatigue on three subscales: physical, cognitive, and psychosocial. Higher total MFIS scores indicate a greater impact of fatigue on a person’s activities. Additional verbally presented neuropsychological tests were used for a correlation analysis with SiN metrics; these were: the PASAT (Gronwall, 1977), Digit Span Test (DST – WAIS-IV administration) and California Verbal Learning Test (CVLT) (Delis et al., 1987).

### Audiometry

Hearing sensitivity was determined using a Beltone Model 110 Clinical Audiometer and calibrated TDH headphones to test sensitivity one ear at a time, at standard audiometric frequencies of 250, 500, 750, 1,000, 1,500, 2,000, 4,000, 6,000, and 8,000 Hz, using a modified Hughson-Westlake procedure (Jerger et al., 1959). Hearing thresholds, recorded as decibels Hearing Level (dB HL) relative to normal sensitivity (ISO 8253-1, 1989), were defined as the lowest level at which the tone was perceived 50% of the time. Pure tone averages (PTAs) of hearing threshold levels at 500, 1000, 2000 and 4000 Hz were obtained for all participants to describe hearing status, and only participants with a bilateral four tone average < 25 dB HL were used in this study. Participants with PTAs ≥ 25 dB were excluded to remove peripheral hearing loss as a confounding factor on speech discrimination ability.

### Speech in Noise Discrimination Tasks

Three speech discrimination tasks were employed in this study: (1) sentences presented in speech-weighted noise (SWN); (2) sentences in multi-talker babble noise (BN) and (3) words in BN. All discrimination tasks were conducted from a Dell Latitude computer, using an in-house program to deliver the sentences and noise at varying SNRs and to store and display data. All auditory stimuli were stored as “.wav” files and presented to participants binaurally through Sennheiser HD535 headphones.

All stimuli was calibrated by coupling the headphones to a Brüel and Kjaer Artificial Ear Type 4152 containing a Brüel and Kjaer 1 Condenser Microphone Type 4145. The microphone output was connected to a Brüel and Kjaer Precision Sound Level Meter Type 2203 from which sound pressure level (SPL) was read directly on an A-weighted scale on ‘slow’ time setting. Sentence levels were calibrated using a reference 1–15 kHz noise band signal with average root mean square level set to the same value as that for the sentences. The noise masker was calibrated by playing the noise through the headphones and using the “slow” time setting to measure output level.

#### Target Speech

Sentences came from the Bamford-Kowal-Bench (BKB) sentence lists for partially hearing children (Bench et al., 1979). The full BKB list contains 192 sentences, each 4–6 words long, with each sentence having three keywords by which identification of the sentence was scored (Bench et al., 1979). Keywords from the BKB sentences were used as stimuli for the words in noise (WiN) task. To ensure the words were presented identically acoustically to how keywords were presented in the sentences in noise task, the words were sliced carefully from the pre-recorded sentences. All target speech was spoken by a female voice with an Australian accent in a neutral tone.

Previous, unpublished work in our laboratory determined psychometric functions for the identification of each of the 192 BKB sentences in SWN (i.e., noise shaped to have energy spread over frequencies as it is for speech). A separate group of 15 normal-hearing participants were tested for the identification of each sentence at a number of SNRs and the SNR at which each fixed-intensity sentence was correctly discriminated 50% of the time was defined as the speech reception threshold (SRT). This SRT was the basis for selection of 120 sentences with similar identifiability and these sentences were then tested and validated against SWN and BN maskers in a large normal-hearing population of different age ranges segregated into decade age groups (Rajan and Cainer, 2008). We have detailed previously our extensive work on the development of our test battery (Burns and Rajan, 2008; Cainer et al., 2008; Rajan and Cainer, 2008). In those papers, we established that for the BKB sentences used in this study, with the same types of background noises as used, discrimination performance asymptotes at approximately 10–15 sentences regardless of noise type and across a wide range of subject ages from 20 to 70 years. In those and in a later paper (Dunlop et al., 2016), we established that a list of 10–15 sentences would suffice to index performance within a session.

#### Masker Noise

Two background noises, SWN and BN were presented to all participants. SWN was shaped to the long-term average spectrum of the target sentences, as measured using a Madsen audiometer. Multi-talker consisted of eight simultaneous voices generated by doubling over and temporally offsetting a recording of four people reading nonsense text. Eight-talker BN was used as it has been shown to be the most effective babble masker for phoneme detection (Simpson and Cooke, 2005). Both noises were digitized and stored as .wav files. The root mean square levels of the two noises were modified to be equal.

#### Speech in Noise Task Procedures

Unique sentences or words were presented one at a time with a background masker and participants were required to verbally repeat each sentence/word they had just heard; or indicate their inability to do so. Participants did not have a motor speech disorder, with the exception of one who had mild dysarthria (which was confirmed by the participant during an informal interview before testing). No time limit was placed on response and feedback was not provided. The experimenter, who was not blinded to group assignment of participants, recorded the responses, and presented the next sentence after 1.5 s delay. All three keywords had to be correctly recalled for a correct response in the sentences in noise task.

Target speech stimuli were always presented at 70 dBA, whilst maskers were presented at a range of levels to generate different SNRs. Sentences were presented at a constant level whilst the masker level was varied to generate SNRs of 1, −1, −3, −5, and −7 dB in SWN and 3, 1, −1, −3, and −5 dB in BN. Prior to each noise condition, participants completed 10 practice trials (10 unique target sentences) at an ‘easy’ SNR of +5 dB for acclimatization to stimuli. Subsequent SNR blocks were presented in random order. Ten unique sentences were presented at each SNR in a randomized order presentation. An identical process was used for the WiN task, however, 30 words were presented at each SNR.

### Loudness Sensitivity Test

Participants were asked to describe the extent of his/her auditory discomfort in response to stimuli presented through headphones. This test of hypersensitivity to sounds was previously conducted in the case of participants with high-functioning Autism Spectrum Disorder (ASD) to interpret difficulties in speech discrimination (Dunlop et al., 2016). We employed a procedure similar to that described by Dunlop et al. (2016).

A chart was placed in front of the participant with the numbers 1–7 drawn in a hemi-circle. Emoticons were placed at the numbers 1, 4, and 7: a smiley face at 1 to indicate no discomfort, a neutral face at 4 to indicate moderate discomfort and a sad face at 7 to indicate great discomfort. Seven sets of three sentences were presented binaurally at levels ranging from 60 dBA (A-weighted decibels) to 90 dBA in 5 dB steps. Sentences were derived from a standard clinically used battery of sentences, the BKB sentence lists consisting of simple sentences in common use (Bench et al., 1979).

BN was also presented binaurally at seven different levels ranging from 65 to 77 dBA in 2 dB steps. BN consisted of eight simultaneous voices generated by doubling over and temporally offsetting a recording of four people reading nonsense text. Participants indicated the extent of auditory discomfort they experienced for each stimulus by pointing to the number that corresponded to their perceived loudness discomfort level. Note: the same BN was used in the speech in discrimination tasks.

### Auditory Attention and Discomfort Questionnaire (AADQ)

The AADQ was developed by Dunlop et al. (2016) and based on validated inventories for specific adult clinical populations experiencing abnormal auditory processing: the Hearing Handicap and Denver Scales (Schow and Nerbonne, 1980), the Hearing Handicap Inventory for the Elderly (Ventry and Weinstein, 1982), the Amsterdam Inventory for Auditory Disability and Handicap (Meijer et al., 2003) and an unpublished inventory developed at The University of Auckland for hearing aid users. The 33-item AADQ consists of statements about daily life events involving hearing and had three subscales; the Audio-Attentional Difficulty subscale measures difficulties attending to speech in noisy environments; the Auditory Discomfort (Non-Verbal) subscale measures discomfort to non-verbal environmental sounds; and the Auditory Discomfort (Verbal) subscale measures discomfort to verbal sounds. Refer to Supplementary Figure A1 for details on the questionnaire.

### Statistical Analyses

Statistical analyses were conducted with IBM SPSS Statistics 26, MATLAB 2019b and GraphPad Prism 8 programs.

Participant demographics and hearing sensitivity were compared across control, early and late mild MS groups by chi-squared tests, Kruskal–Wallis Tests and One-Way ANOVAs, depending on the distribution of data sets.

Depression, fatigue, premorbid intelligence levels and neuropsychological evaluations were compared between early and late mild MS groups by Mann–Whitney or unpaired Student’s *t*-tests, again depending on the distribution of data sets.

Pure-tone hearing thresholds and all SiN tasks were evaluated using two-way mixed-effects analysis of variance (ANOVA) and *post hoc* Tukey’s multiple comparisons tests. Boltzmann sigmoidal functions were fitted to obtain psychometric curves as a function of SNR for individual participants in each SiN task. Slope and midpoint data from the curves were compared using one-way ANOVAs.

Pearson’s correlations were used to determine the relationship between SiN measures and several clinical and neuropsychological measures; with the exception of the association between EDSS scores and SiN measures, which was run as a Spearman correlation.

The midpoints of the psychometric curve for each SiN task were used in analyses of receiver operating characteristic curves to classify between controls and all pwMS. Areas under the curves (AUC-ROC) were obtained to evaluate classification performance. Youden’s Index was used to determine a cut-off point, and sensitivity and specificity were obtained.

A logistic regression model was developed to discriminate between controls (coded as 0) and all low impaired MS participants (coded as 1). The model building strategy was to only consider speech discriminated at certain SNRs as predictor variables. The model was validated using fivefold cross validation, AUC-ROC and confusion matrix.

## Results

### Participant Demographics, Characteristics, and Audiometric Hearing Status

50 controls and 40 pwMS were recruited for this study, however, 12 controls (24%) and 5 pwMS (12.5%) were excluded for bilateral hearing loss (PTAs ≥ 25 dB HL). The remaining 38 controls and 35 pwMS (Table 1) had bilaterally normal hearing between 250 and 4000 Hz; of these, 5% from each group had small hearing losses (of 5–10 dB) at the higher test frequencies of 6000 and 8000 Hz in one ear only. Of the 35 pwMS, 15 relapsing-remitting and 8 CIS participants were classified as early mild-MS (≤5 years after diagnosis; EDSS < 3) and 12 relapsing-remitting as late mild-MS (≥10 years after diagnosis; EDSS < 3).

**TABLE 1:** Participant demographics, auditory evaluation, disease characteristics, estimated premorbid intelligence, depression, fatigue, and neuropsychological test details.

		Control	All MS	Early MS	Late MS	*p*-value
**Demographics**	**Number of participants**	38	35	23	12	
	**Sex F(*M*)**	35 (3)	31(4)	20(3)	11(1)	
	**Age, (years)**					
	Mean (*SD*)	45.66 (10.43)	44.94 (10.59)	42.86 (11.10)	49.25 (8.32)	0.22**^a^**
	Range	28 – 60	26 – 65	26 – 65	37 – 65	
**Auditory evaluation**	**Pure tone average (dB HL)**					
	Left (Mean, *SD*)	13.03(4.80)	12.79(4.34)	12.78(4.68)	12.82(3.81)	0.98**^b^**
	Right (Mean, *SD*)	11.97(4.49)	13.11(5.08)	13.26(4.98)	12.81(5.49)	0.58**^b^**
	**Auditory Attention and Distress Questionnaire**					
	Audio-attentional difficulty (Mean, SD) Total/98*	23.63 (8.11)	25.86 (11.13)	24.61(10.33)	28.25(12.63)	0.36**^b^**
	Auditory Discomfort (non-verbal)(Mean, SD) Total/56*	26.05 (9.29)	24.06 (8.27)	23.30 (8.59)	25.50 (7.76)	0.50**^b^**
	Auditory Discomfort (verbal) (Mean, SD) Total/35*	16.79 (6.34)	17.80 (8.05)	16.61 (6.80)	20.08 (9.95)	0.34**^b^**
**Diseasecharacteristics**	**Disease duration (years)**					
	Mean (*SD*)	NA	7.26 (6.25)	3.14 (1.59)	14.8 (3.63)	**<0.0001^c^**
	Range	NA	0 – 22	0 – 5	10 – 22	
	**EDSS∼**					
	Mean (*SD*)	–	0.37(0.81)	0.35 (0.82)	0.42 (0.82)	0.69**^c^**
	Range	–	0 – 2.5	0 – 2.5	0 – 2.5	
	**Phenotype RR(CIS)**	NA	27(8)	15(8)	12(0)	
	**On disease modifying therapy (n, %)**	NA	29 (82.86%)	19 (82.61%)	10 (83.33%)	0.99**^d^**
**EstimatedPremorbidIntelligence**	**National Adult Reading Test**					
	Mean (*SD*)	–	116(5.31)	114.9(5.18)	117(5.61)	0.36**^e^**
	Range	–	105 – 125	105 – 124	105 – 125	
	Data missing (n,%)	–	9 (25.71%)	6 (26.09%)	3 (25%)	
**Depression**	**Beck’s Depression Index**					
	Mean (*SD*)	–	4.79(4.43)	4.9(4.32)	4.6(4.9)	0.85**^e^**
	Range	–	0–14	0 – 14	0–13	
	Data missing (*n*, %)	–	6 (17.14%)	4 (17.39%)	2 (16.67%)	
**Fatigue**	**Modified Fatigue Impact Scale**					
	Mean (*SD*)	–	26.25(16.08)	28.58(15.87)	21.33(16.31)	0.15**^c^**
	Range	–	0–49	0–49	0–39	
	Data missing (*n*, %)	–	7 (20%)	4 (17.39%)	3 (25%)	
**Neuropsychologicalassessments(delivered in theauditory domain)**	**Paced Auditory Serial Addition Test**					
	Mean % (*SD*)	–	82.83(19.62)	85.87(16.45)	77.03(24.48)	0.35**^c^**
	Range	–	28.33 – 100	51.67 – 100	28.33–100	
	Data missing (*n*, %)	–	6 (17.14%)	4 (17.39%)	2 (16.67%)	
	**California Verbal Learning Test**					
	Mean (*SD*)	–	45.44(14.03)	42.47(13.7)	50.5(13.79)	0.15**^e^**
	Range	–	10–67	10–62	19–67	
	Data missing (*n*, %)	–	6 (17.14%)	4 (17.39%)	2 (16.67%)	
	**Digit Span (Forward)**					
	Mean (*SD*)	–	11.04(2.68)	11.44(2.78)	10.12(2.36)	0.25**^e^**
	Range	–	6 – 16	7 - 16	6 – 14	
	Data missing (*n*, %)	–	9 (25.71%)	5 (21.74%)	4 (33.33%)	
	**Digit Span (Backward)**					
	Mean (*SD*)	–	7.27(2.19)	7.5(2.12)	6.75(2.49)	0.44**^e^**
	Range	–	3 – 11	4 – 11	3 – 10	
	Data missing (*n*, %)	–	7 (20%)	5 (21.74%)	2 (16.67%)	

F; female; M, male; dB HL, decibels hearing level; RR, relapsing remitting; CIS, clinically isolated syndrome; SD, standard deviation; NA, not applicable; n, number of participants; ∼EDSS, Expanded Disability Status Scale Score determined by a neurologist within 6 months of audiological testing. *Higher total scores in the AADQ were indicative of greater audio-attentional difficulty (range 14–98); greater non-verbal discomfort (range 8–56); and greater verbal discomfort (range 5–35). ^a^Kruskal–Wallis Test ^b^One-way ANOVA ^c^Mann–Whitney Test ^d^Fisher’s Test ^e^Unpaired Student’s t-test Demographics and audiometry metrics were compared across controls, early, and late mild MS. Disease characteristics, estimated premorbid intelligence, fatigue, and neuropsychological assessments were compared between early and late mild MS. p values in bold represent significant differences between groups (p < 0.05).

**FIGURE 1:**
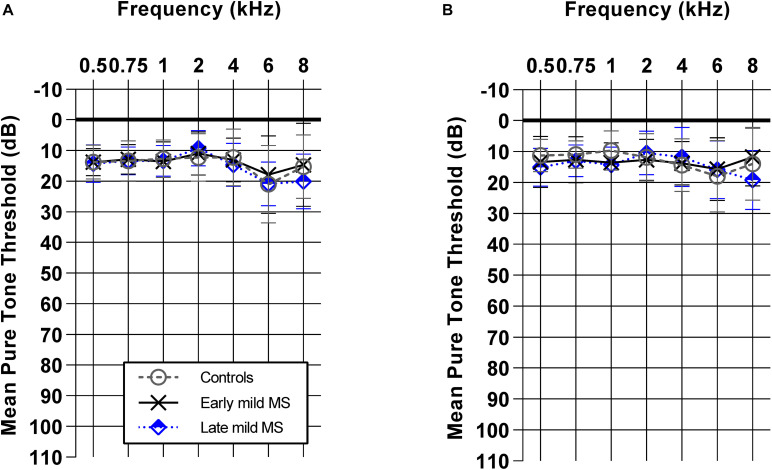
Early and late mild MS groups have similar pure-tone hearing sensitivity (0.5–8 kHz) to controls. Mean pure tone thresholds [± standard deviation (SD)] obtained from left **(A)** and right **(B)** ears of control (*n* = 38; circle/solid line), early mild-MS (*n* = 23; cross/broken line) and late mild-MS (*n* = 12; diamond/dotted line) were not significantly different. Two-way mixed ANOVA (*p* > 0.05).

Demographics such as age, sex, and mean pure-tone averages (dB HL) did not differ between controls, early mild MS, and late mild MS groups (*p* > 0.05). Figure 1 shows mean pure tone air-conduction thresholds at audiometric test frequencies for left (Figure 1A) and right (Figure 1B) ears of the three groups. A two-way mixed-effects analysis of variance (ANOVA) confirmed that hearing sensitivity was similar for the three groups for both left [*F*(2,70) = 0.29, *p* = 0.75, η^2^ = 0.26] and right ears [*F*(2,70) = 0.39, *p* = 0.68, η^2^ = 0.34]. In summary, the three groups all had normal hearing that was similar across groups.

With respect to the two MS sub-groups, early mild-MS and late mild-MS, disease duration was statistically significant (*p* < 0.0001) but EDSS scores and the percentage of participants on disease modifying therapies were not statistically significant. There were also no differences in performance on the neuropsychological and cognitive test results. Estimated premorbid intelligence, depression, and fatigue (as measured by the NART, BDI, and MFIS scores, respectively) were not statistically significant between the early and late mild-MS groups. Performance on tests involving auditory input, such as the PASAT, CVLT, and digit span tests (forward and backward), also did not differ statistically between early and late mild-MS groups. Thus, although the early mild-MS and late mild-MS sub-groups differed in disease duration, they did not differ on EDSS scores and neuropsychological tests, including tests with an auditory processing component.

### Sensory Discomfort to High Intensity Stimuli

**FIGURE 2:**
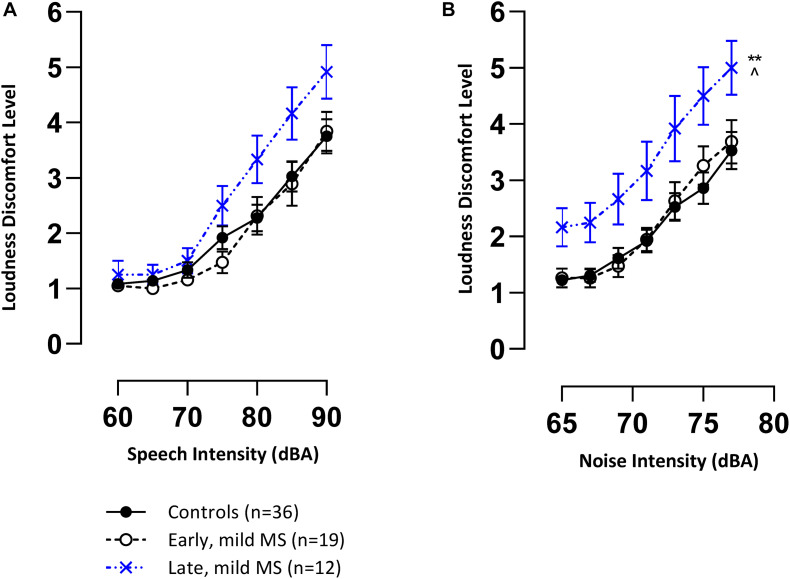
Late mild pwMS have higher discomfort levels to speech and noise (multi-talker babble) stimuli compared to early mild pwMS and controls. Mean loudness discomfort levels (±SEM) of control (*n* = 36; filled circle/solid line), early mild-MS (*n* = 19; empty circle/broken line) and late mild-MS (*n* = 12; cross/dotted line) for speech stimuli **(A)** and multi-talker babble **(B)** presented at various intensities (A-weighted decibels). **p < 0.01 compared to controls; ^*p* < 0.05 compared to early mild MS. Two-way mixed ANOVA, and Tukey’s *post hoc* test (adjusted *p*-value > 0.05).

Mean loudness discomfort levels (LDLs) (±SEM) for speech and BN are represented in Figures 2A,B, respectively. The trend in both graphs demonstrate that controls and pwMS were, on average, increasingly uncomfortable as speech and noise stimuli were presented at louder intensities (dB) (1 = comfortable to 7 = discomfort). This trend was confirmed by 3 × 7 two-way mixed ANOVAs which revealed an effect of intensity (dB) on LDLs for speech [*F*(6,384) = 115.5, *p* < 0.0001, η^2^ = 39.03] and noise [*F*(6,384) = 106.7, *p* < 0.0001, η^2^ = 25.33].

An interaction effect between disease group and intensity on speech LDLs was significant [*F*(12,384) = 1.89, *p* = 0.03]. This result and the trend in Figure 2A suggest that late pwMS reported higher LDLs than early pwMS and controls only at higher speech intensities. A Tukey’s multiple comparisons test revealed that there was no statistical difference at any of the seven intensity levels between disease groups (adjusted *p*-value > 0.05).

There was no significant interaction effect between disease group and intensity on noise LDLs [*F*(12,384) = 0.86, *p* = 0.59], but there was a significant effect of disease group [*F*(2,64) = 5.35, *p* = 0.007]. A Tukey’s multiple comparisons test confirmed that late mild pwMS had significantly higher noise LDLs compared to early mild pwMS (adjusted *p*-value = 0.02) and controls (adjusted *p*-value = 0.006) (Figure 2B). All pwMS were tested > 3 months after a relapse of symptoms, hence, sensitivity to sound was unlikely to be a transient episode of hyperacusis which has been reported in rare case reports (Weber et al., 2002).

### Discrimination of Sentences in Noise

**FIGURE 3:**
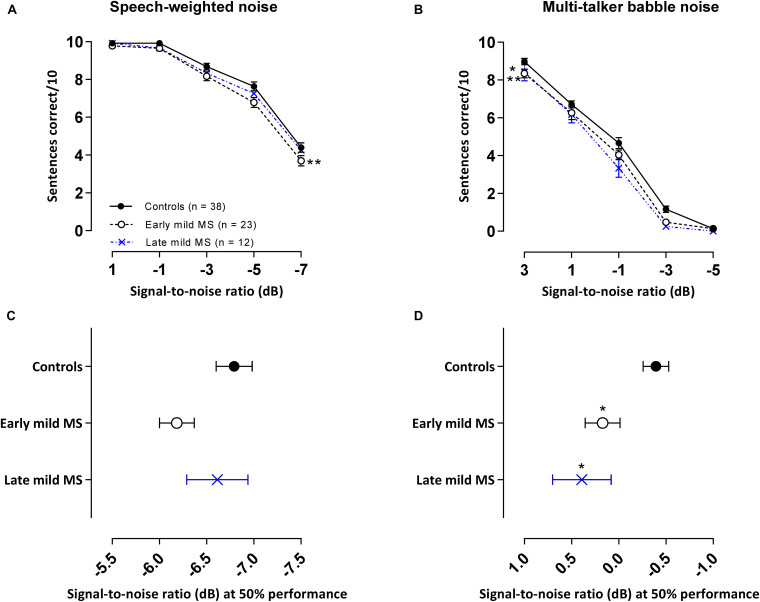
PwMS discriminated fewer sentences than controls in an attention-demanding babble masker, but not in speech-weighted noise. Mean ± SEM correctly discriminated sentences (i.e., all three keywords detected in a sentence) out of 10 test sentences at each signal-to-noise ratio [SNR (dB)] for control (*n* = 38, filled circles/solid line), early (*n* = 23, open circle/dashed line), and late mild MS (*n* = 12, cross/dotted line) in speech-weighted noise **(A)** and multi-talker babble **(B).** Mean SNRs ± SEM (dB) at 50% discrimination in speech-weighted noise **(C)** and multi-talker babble **(D)** were calculated from the midpoints of Boltzmann sigmoidal fitting functions. *p < 0.05; **p < 0.01; compared to controls. (**A,B**: mixed-effects two-way ANOVA; **C,D:** one-way ANOVA; all cases Tukey’s *post hoc* test).

In our core tasks, participants were tested with different lists of sentences separately in SWN and in BN. As shown in Figures 3A,B, the general trend for all listeners in both conditions was a decrease in sentence identification as SNR decreased. SiN discrimination was easier in SWN than BN as controls were able to correctly identify 50% of the sentences at an SNR of −6.8 ± 0.19 dB in SWN compared to a higher SNR of −0.39 ± 0.38 dB in BN for the same level of performance.

#### Discrimination of Sentences in Speech-Weighted Noise

Effects in the energetic SWN masker are shown in Figure 3A. Sentence discrimination in SWN was relatively easy for SNRs ≥ −1 dB at which controls and mild-MS participants had close to perfect performance recall (98.1%) but at SNRs < −1 dB sentence discrimination degraded for all listeners. These effects were confirmed by a 3 × 5 [i.e., 3 treatment groups (control, early mild-MS, late mild-MS) × 5 SNRs (1, −1, −3, −5, and −7 dB)] two-way mixed ANOVA. No interaction effects were significant between SNR and listener group [*F*(8,280) = 0.70, *p* = 0.70, η^2^ = 0.27], but there was a significant main effect of listener group [*F*(2,70) = 4.86, *p* = 0.01, η^2^ = 0.85] and of SNR [*F*(4,280) = 328.8, *p* < 0.0001, η^2^ = 64.02]. Simple main effects analysis showed that early mild pwMS discriminated fewer sentences than controls (adjusted *p*-value = 0.008), but there were no differences between late mild-MS and controls (adjusted *p*-value = 0.24) or late mild-MS and early mild-MS (adjusted *p*-value = 0.43).

#### Discrimination of Sentences in Multi-Talker Babble

Effects in the attentionally demanding BN are shown in Figure 3B. The BN appeared to degrade speech intelligibility for mild-MS listeners more than controls at all SNR conditions except at an SNR of −5 dB at which a floor effect was observed for all groups. A 3 × 5 (i.e., 3 treatment groups × 5 SNRs) two-way mixed ANOVA was used to compare the ability of the groups to discriminate sentences in BN in various SNR conditions. No interaction effects were found [*F*(8,280) = 1.07, *p* = 0.38, η^2^ = 0.23], however, there was a significant main effect of listener group [*F*(2,70) = 6.29, *p* = 0.003, η^2^ = 0.66] and SNR [*F*(4,280) = 668.8, *p* < 0.0001, η^2^ = 70.6]. Simple main effects analysis showed that both early and late mild-MS groups discriminated fewer sentences than controls (adjusted *p*-value = 0.03 and adjusted *p*-value = 0.009, respectively). There was no difference in discrimination between early and late mild-MS groups (adjusted *p*-value > 0.99).

To quantify MS effects on sentence discrimination, Boltzmann sigmoidal functions were fitted to each participant’s discrimination curves, with the top and bottom of the functions constrained to 10 and 0 sentences correct, respectively. From each such psychometric curve, the slope and midpoint SNR were extracted (see Table 2). Measures of goodness of fit were strong for each group (*R*^2^ always > 0.9). A one-way ANOVA revealed no significant difference in mean slopes of the psychometric functions for control, early, and late mild-MS groups for speech discrimination in SWN [*F*(2,70) = 1.10, *p* = 0.35, η^2^ = 0.03] or in BN [*F*(2,70) = 0.50, *p* = 0.61, η^2^ = 0.01].

**TABLE 2:** Degrees of freedom (df), goodness of fit (R^2^), slope ± SE, and midpoint ± SE values for Boltzmann sigmoidal functions fitted to the mean sentences correctly discriminated at tested signal-to-noise-ratios.

		df	*R*^2^	Midpoint ± SE SNR (dB)	Slope ± SE (Sentences/dB)
**Speech-weighted noise**	**Controls**	37	0.91	−6.79 ± 0.19	1.54 ± 0.13
	**Early mild MS**	22	0.93	−6.19 ± 0.18	1.78 ± 0.14
	**Late mild MS**	11	0.92	−6.61 ± 0.32	1.85 ± 0.23
**Multi-talker babble noise**	**Controls**	37	0.92	−0.39 ± 0.13	1.43 ± 0.08
	**Early mild MS**	22	0.93	0.17 ± 0.19*	1.52 ± 0.09
	**Late mild MS**	11	0.93	0.39 ± 0.31*	1.36 ± 0.14

df, degrees of freedom; R^2^, goodness of fit; SNR, signal-to-noise ratio; SE, standard error; dB, decibels. *p < 0.05; compared to control in babble, one-way ANOVA with Tukey’s multiple comparisons post hoc test. p values in bold represent significant differences between groups (p < 0.05).

The mean midpoint SNRs ± SEM (dB) of the curves are graphed in Figures 3C,D for sentence discrimination in SWN and BN, respectively (refer to Supplementary Figure A2 for scatterplots of individual participant midpoint SNRs). A one-way ANOVA indicated that the midpoint SNRs of control, early, and late mild-MS psychometric functions for sentence discrimination in SWN were not significantly different [*F*(2,70) = 2.25, *p* = 0.11, η^2^ = 0.06]. In contrast, midpoint SNRs of control, early, and late mild-MS psychometric functions for speech discrimination in BN were significantly different [*F*(2,70) = 4.9, *p* = 0.01, η^2^ = 0.12] A Tukey’s multiple comparisons test confirmed that early mild and late-mild MS participants required significantly higher SNRs for 50% discrimination accuracy compared to controls (adjusted *p*-value = 0.04 and 0.02, respectively). There was no significant difference between the SNRs of the midpoints of the curves for the two MS groups (adjusted *p*-value = 0.76).

At the SNR (−0.39 ± 0.13 dB) at which controls attained 50% sentence intelligibility in BN, speech intelligibility in early mild pwMS and late mild pwMS was 9.11 ± 0.21% and 13.96 ± 0.22% lower, respectively.

### Discrimination of Keywords in Noise

In the SiN tasks detailed above, especially at lower SNRs, listeners were often able to identify some of the words in a sentence but not all three keywords required to score correct discrimination of the whole sentence. We therefore conducted a second analysis where we examined the number of keywords detected correctly across all 10 sentences (30 keywords total at 3/sentence) for each SNR block in the SiN task whether the sentence in which the keyword was embedded was scored correct or not.

#### Discrimination of Keywords in Speech-Weighted Noise

**FIGURE 4:**
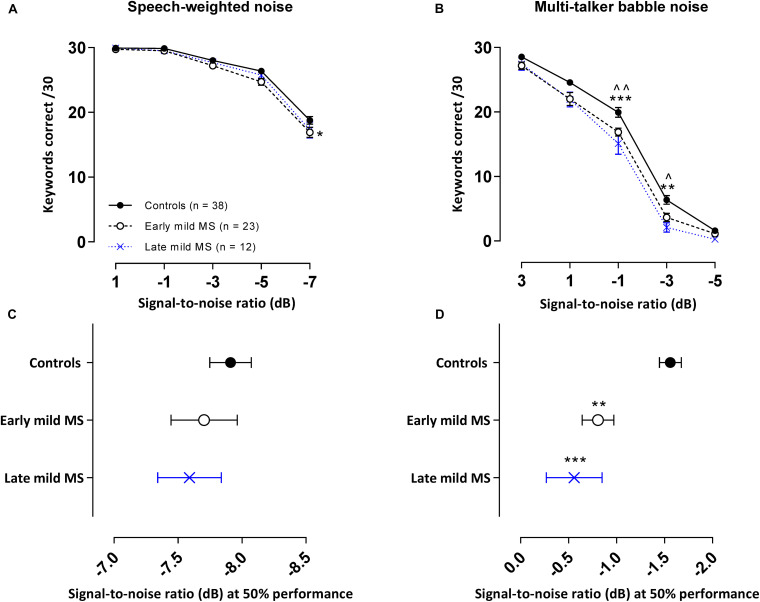
PwMS discriminated fewer keywords than controls in an attention-demanding informational babble masker. Mean ± SEM keywords {out of 30 at each signal-to-noise ratio [SNR (dB)]} correctly discriminated for control (*n* = 38, filled circles/solid line), early (*n* = 23, open circle/dashed line), and late mild-MS (*n* = 12, cross/dotted line) in speech-weighted noise **(A)** and multi-talker babble **(B).** Mean SNRs ± SEM (dB) at 50% discrimination in speech-weighted noise **(C)** and multi-talker babble **(D)** were calculated from the midpoints of Boltzmann sigmoidal fitting functions. (*)(^)*p* < 0.05; (**)(^^)*p* < 0.01; ***p < 0.001; compared to controls. (**A,B**: mixed-effects two-way ANOVA; **C,D:** one-way ANOVA; all cases Tukey’s *post hoc* test).

The mean number of keywords (±SEM) correctly discriminated by controls, early and late mild MS listeners in each SNR block in the SWN masker is presented in Figure 4A. A two-way mixed ANOVA indicated that there was no interaction effect between SNR and listener group [*F*(8,280) = 1.04, *p* = 0.41, η^2^ = 0.43], but there was a significant main effect of listener group [*F*(2,70) = 4.61, *p* = 0.01, η^2^ = 0.78] and of SNR [*F*(4,280) = 322.9, *p* < 0.0001, η^2^ = 66.2]. Simple main effects analysis showed that early mild-MS participants discriminated fewer sentences than controls (adjusted *p*-value < 0.05), but there were no differences between late mild-MS and controls or late mild-MS and early mild-MS (adjusted *p*-value > 0.05).

#### Discrimination of Keywords in Multi-Talker Babble

The mean number of keywords (±SEM) correctly discriminated by controls, early and late mild MS listeners in each SNR block in the BN masker is presented in Figure 4B. A two-way mixed ANOVA indicated the presence of a significant interaction effect between SNR and listener group [*F*(8,280) = 2.22, *p* = 0.03, η^2^ = 0.31], as well as a significant main effect of listener group [*F*(2,70) = 11.40, *p* < 0.0001, η^2^ = 1.18] and SNR [*F*(4,280) = 1031, *p* < 0.0001, η^2^ = 72.91]. Simple effects analysis showed that early, and late mild-MS participants discriminated fewer sentences than controls at an SNR of −1 dB (adjusted *p*-value = 0.007 and adjusted *p*-value = 0.0002, respectively) and at an SNR of −3 dB (adjusted *p*-value = 0.03 and adjusted *p*-value = 0.001, respectively). There was no significant difference in discrimination between early and late mild-MS groups at either of these SNRs (adjusted *p*-value > 0.05).

To quantify MS effects on keyword discrimination, Boltzmann sigmoidal functions were fitted to each participant’s discrimination curves. The top and bottom of the functions were constrained to 30 and 0 keywords correct respectively. Measures of goodness of fit were strong for each group (*R*^2^ ≥ 0.9). Table 3 displays the slope and midpoint data extracted from the psychometric curves.

**TABLE 3:** Degrees of freedom (df), goodness of fit (R^2^), slope ± SE, and midpoint ± SE values for Boltzmann sigmoidal functions fitted to the mean keywords correctly discriminated at tested signal-to-noise-ratios in speech-weighted and babble noise.

		df	*R*^2^	Midpoint ± SE SNR (dB)	Slope ± SE (Keywords/dB)
**Speech-weighted noise**	**Controls**	37	0.93	−7.91 ± 0.16	1.51 ± 0.11
	**Early mild MS**	22	0.94	−7.70 ± 0.25	1.79 ± 0.18
	**Late mild MS**	11	0.95	−7.59 ± 0.25	1.50 ± 0.18
**Multi-talker babble noise**	**Controls**	37	0.96	−1.56 ± 0.11	1.18 ± 0.06
	**Early mild MS**	22	0.94	−0.81 ± 0.16**	1.40 ± 0.10
	**Late mild MS**	11	0.95	−0.56 ± 0.29***	1.18 ± 0.11

df, degrees of freedom; R^2^, goodness of fit; SNR, signal-to-noise ratio; SE, standard error; dB, decibels. **p < 0.01; ***p < 0.001, compared to controls in babble, one-way ANOVA with Tukey’s multiple comparisons post hoc test. p values in bold represent significant differences between groups (p < 0.05).

A one-way ANOVA revealed no significant difference in mean slopes of control, early, and late mild-MS psychometric functions for keyword discrimination in SWN [*F*(2,69) = 1.27, *p* = 0.29, η^2^ = 0.04] or BN [*F*(2,70) = 3.14, *p* = 0.051, η^2^ = 0.08]. The mean midpoint SNRs ± SEM (dB) of the curves are graphed in Figures 4C,D for discrimination in SWN and BN, respectively (refer to Supplementary Figure A3 for scatterplots of individual participant midpoint SNRs). A one-way ANOVA indicated that there was no significant difference in mean midpoints of control, early, and late mild-MS psychometric functions for keyword discrimination in SWN [*F*(2,69) = 0.53, *p* = 0.59, η^2^ = 0.02].

In contrast, there was a significant difference in mean midpoints of control, early, and late mild-MS psychometric functions for keyword discrimination in BN [*F*(2,70) = 10.84, *p* < 0.0001, η^2^ = 0.24]. A Tukey’s multiple comparisons test indicated that early and late mild-MS participants needed significantly higher SNRs to achieve 50% correct performance compared to controls (adjusted *p*-value = 0.002 and 0.0007, respectively). There was no significant difference between the midpoints of the curves for early and late mild MS participants (adjusted *p*-value = 0.65).

At the same SNR (−1.56 ± 0.11 dB) at which controls attained 50% keyword intelligibility in BN, intelligibility was 13.08 ± 0.54% and 20.00 ± 0.55% lower for early mild pwMS and late mild pwMS, respectively.

### Words in Babble Discrimination

Sentences provide additional syntactic and semantic cues that the listener can use to infer the meaning of partially masked or degraded speech (Heinrich and Knight, 2016). For this reason, SiN tasks often employ simpler stimuli, like phonemes or isolated WiN to assess speech discrimination ability; despite the use of sentences reflecting better communication demands in the real listening world. We therefore examined whether the effects seen above with whole sentences or with keywords embedded in sentences would be replicated with isolated words, in a background of BN. For this task, individual keywords were extracted from the pre-recorded sentences of the BKB sentence lists used above, and were presented individually in random order (thereby removing any linguistic context) at similar SNRs as used in the SiN task with the same BN (3, 1, −1, −3, −5 dB).

**FIGURE 5:**
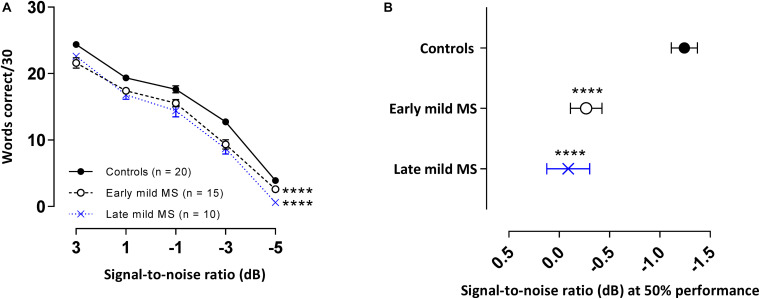
PwMS discriminated fewer words in multi-talker babble compared to healthy controls. Mean ± SEM words [out of 30 at each signal-to-noise ratio (SNR)] correctly discriminated for control (*n* = 20, filled circles/solid line), early (*n* = 15, open circle/dashed line), and late mild MS (*n* = 10, cross/dotted line) in multi-talker babble **(A).** Mean SNRs ± SEM (dB) at 50% discrimination in multi-talker babble were calculated from the midpoints of Boltzmann sigmoidal fitting functions **(B)**. ****p < 0.0001; compared to controls. (**A:** mixed-effects two-way ANOVA; **B:** one-way ANOVA; all cases Tukey’s *post hoc* test).

Not all participants were able to attend the additional session where this test was conducted and so 20 controls, 15 early, and 10 late mild MS participants completed the WiN task. The means and standard errors of correctly recalled words in BN for controls and MS participants are presented in Figure 5A. A 3 × 5 two-way mixed ANOVA revealed main group effects were significant [*F*(2,49) = 26.96, *p* < 0.0001, η^2^ = 3.10]. Additionally, as expected, a decrease in SNR significantly negatively impacted word discrimination in all listener types [*F*(4,196) = 656.0, *p* < 0.0001, η^2^ = 76.2]. Decreasing SNR degraded speech discrimination performance similarly for all listeners, as evident by no interaction effect [*F*(8, 196) = 1.50, *p* = 0.16, η^2^ = 0.35]. Simple main effects analysis showed that both early and late mild-MS participants discriminated fewer sentences than controls (adjusted *p*-value < 0.0001). There was no difference in discrimination between early and late mild-MS groups (adjusted *p*-value = 0.50).

Again, to quantify MS effects on word discrimination, Boltzmann sigmoidal functions were fitted to each participant’s discrimination curves. Table 4 displays the slope and midpoint data extracted from the psychometric curves. The top and bottom of the functions were constrained to 30 and 0 words correct, respectively. Measures of goodness of fit were strong for each group (*R*^2^ ≥ 0.88). A one-way ANOVA confirmed no significant difference in mean slopes of psychometric functions for control, early, and late mild-MS groups for word discrimination in BN [*F*(2,42) = 3.03, *p* = 0.06, η^2^ = 0.13]. In contrast, there was a significant difference in mean psychometric function midpoints [*F*(2,42) = 16.55, *p* < 0.0001, η^2^ = 0.44]. The mean midpoint SNRs ± SEM (dB) of the curves are graphed in Figure 5B (refer to Supplementary Figure A4 for scatterplots of individual participant midpoint SNRs). A Tukey’s multiple comparisons test indicated that the midpoints of the curves for early and late mild-MS participants were at significantly higher SNRs compared to those for controls (adjusted *p*-value < 0.0001). There was no significant difference between the midpoints of the curves of early and late mild-MS participants (adjusted *p*-value = 0.76). Thus, these data showed that the psychometric functions for the early and late mild-MS groups had shifted to the left.

**TABLE 4:** Degrees of freedom (df), goodness of fit (R^2^), slope ± SE, and midpoint ± SE values for Boltzmann sigmoidal functions fitted to the mean words correctly discriminated at tested signal-to-noise-ratios in babble noise.

	Df	*R*^2^	Midpoint ± SE SNR (dB)	Slope ± SE Words/dB
**Controls**	19	0.90	−1.24 ± 0.13	2.91 ± 0.10
**Early mild MS**	14	0.88	−0.27 ± 0.16****	3.06 ± 0.19
**Late mild MS**	9	0.88	−0.09 ± 0.21****	2.53 ± 0.07

df, degrees of freedom; R^2^, goodness of fit; SNR, signal-to-noise ratio; SE, standard error; dB, decibels. ****p < 0.001, compared to controls in babble, one-way ANOVA with Tukey’s multiple comparisons post hoc test. p values in bold represent significant differences between groups (p < 0.05).

At the same SNR (−1.24 ± 0.13 dB) at which controls attained 50%-word intelligibility in BN, intelligibility was 7.86 ± 0.32% and 11.17 ± 0.38% lower in early mild pwMS and late mild pwMS, respectively.

Across the three discrimination in BN tasks (sentences, keywords, and word discrimination), the similarity of slopes across all three groups showed that any changes across groups were not in the shape of the curves but in the curve location along the SNR axis. This was confirmed by the differences in curve midpoints; i.e., mild MS participants needed more favorable SNRs to achieve the same level of performance. The effect sizes (η^2^) for differences in curve midpoints for sentence, keyword and word discrimination in BN were 0.12, 0.24, 0.44, respectively (i.e., 12, 24, and 44% of the total variance accounted for by the group). Comparison of the effect sizes showed that this shift was most pronounced for the isolated words (absent contextual cues), less so for the keywords embedded in sentences, and least when full contextual and semantic cues were present in the whole sentences.

Our analyses thus far have shown clearly that although early and mild late-MS participants differed significantly with regard to duration of disease, they did not differ significantly on any other metric of the disease or any of the speech performance measures or the neuropsychological tests. Thus, for these groups of pwMS, the determining characteristic appeared to be the fact that they had mild MS. Hence for all subsequent analyses, we pooled these two sub-groups of mild-MS participants into a single pool of people with mild MS.

### Correlations of Speech Discrimination Measures to Standard Neuropsychological Tests

Most of our pwMS group underwent neuropsychological testing according to standardized instructions (refer to Table 1 for missing data details). We compared performance on these tests against SiN performance, indexing the latter using the midpoints of the psychometric curves (i.e., SNR at 50% speech intelligibility) since that metric had differed significantly from control values (for sentence or word tasks in BN) whereas the slopes of the psychometric functions had not. A Pearson product-moment correlation coefficient was computed to assess the relationship between performance (out of 100%) in the neuropsychological assessments and the midpoints of the psychometric curves, whilst Spearman correlation coefficients were determined for the relationship between EDSS scores and SiN measures. Correlation coefficients (*r*) between clinical and discrimination measures are displayed in Table 5. For all three types of speech stimuli (sentences, keywords, and words) tested in BN there was a significant negative association between PASAT scores and the midpoints of the psychometric functions (poorer performance on the PASAT related to poorer performance on the SiN task). There was also a significant negative association between the CVLT scores and the midpoints of the psychometric functions. No significant correlations were found between any clinical measures and the midpoints of the psychometric functions for any speech discrimination tests in SWN. No significant relationships were observed between any of the speech discrimination measures and EDSS, disease duration or digit span tests (forward and backward).

**TABLE 5:** Correlation coefficients between neuropsychological/clinical measures and speech-in-noise measures [r, (rs^), 95% confidence intervals] in all mildly impaired MS participants.

	SWN		BN		
	*Sentences*	*Keywords*	*Sentences*	*Keywords*	*Words*
Age (years)_	−0.22 (−0.51 to 0.12)	−0.30 (−0.57 to 0.03)	0.19 (−0.15 to 0.49)	0.07 (−0.26 to 0.39)	−0.17 (−0.52 to 0.24)
Disease Duration (yrs)	−0.19 (−0.49 to 0.16)	0.03 (−0.31 to 0.36)	0.15 (−0.19 to 0.46)	0.13 (−0.21 to 0.45)	0.14 (−0.26 to 0.50)
EDSS^	0.32 (−0.03 to 0.59)	0.00 (−0.33 to 0.33)	0.23 (−0.11 to 0.53)	0.12 (−0.22 to 0.44)	0.12 (−0.3 to 0.50)
BDI	0.00 (−0.36 to 0.36)	0.05 (−0.32 to 0.40)	0.09 (−0.28 to 0.44)	0.07 (−0.30 to 0.42)	0.30 (−0.03 to 0.57)
NART	−0.25 (−0.57 to 0.15)	−0.21 (−0.55 to 0.19)	−0.32 (−0.63 to 0.07)	−0.32 (−0.63 to 0.07)	−0.33 (−0.67 to 0.13)
CVLT	−0.24 (−0.57 to 0.14)	−0.25 (−0.57 to 0.14)	−0.55** (−**0.77 to**−**0.21**)	−0.39* (−**0.67 to**−**0.01**)	−0.44* (−**0.73,**−**0.01**)
Digit Span (Forward)	−0.15 (−0.51, 0.24)	−0.12 (−0.48, 0.28)	−0.19 (−0.54, 0.21)	−0.23 (−0.56, 0.17)	−0.29 (−0.63, 0.16)
Digit Span (Backward)	−0.13 (−0.48, 0.27)	−0.01 (−0.39, 0.38)	−0.31 (−0.62, 0.08)	−0.38 (−0.66, 0.01)	−0.38 (−0.66, 0.01)
PASAT	−0.02 (−0.38, 0.34)	−0.28 (−0.58, 0.09)	−0.58*** (−**0.78,**−**0.27**)	−0.43* (−**0.68,**−**0.08**)	−0.38 (−0.69, 0.02)

SWN, speech-weighted noise; BN, babble noise; EDSS, Expanded Disability Status Scale; BDI, Beck’s Depression Index; NART, National Adult Reading Test; CVLT, California Verbal Learning Test; PASAT, Paced Auditory Serial Addition Test. ^Correlation values are Pearson correlations coefficients, with the exception of the associations between EDSS and SiN measures, which are expressed as Spearman correlation coefficients. *p < 0.05; **p < 0.01; ***p < 0.001. p values in bold represent significant differences between groups (p < 0.05).

### Comparison of All SiN Measures to Discriminate Between Controls and All MS Group

Receiver operating characteristic curves (ROC) were used to evaluate the ability of SiN measures (SNR at 50% discrimination) to discriminate between healthy controls and all pwMS. Table 6 shows the number of observations obtained for each task, time to administer the test, the cut off SNR point (Youden’s *J* statistic) at which participants were categorized as control or MS, sensitivity (true positive rate), specificity (true negative rate) and the area under the curve (AUC). Interpretation of the AUC ROC indicates that sentence and keyword discrimination in SWN was not useful in discriminating between control and MS participants. In contrast, keyword discrimination in BN was acceptable (0.7 – 0.8). Word discrimination in BN provided outstanding discrimination ability (>0.9) (Hosmer, 2000).

**TABLE 6:** The utility of SiN tasks in discriminating between controls and all mild MS: details of the receiver operating characteristic curves.

	Number of observations	Time (min) to administer test	Cut-off SNR (dB) at 50% discrimination^	Sensitivity (%)	Specificity (%)	ROC AUC (95% C.I)
**(1) Sentences in…**						
(a) SWN	73	10	−6.33	51.43	71.05	0.62 (0.49 – 0.75)
(b) BN	73	10	−0.75	94.29	39.47	0.69 (0.56 – 0.81)*
**(2) Keywords in…**						
(a) SWN	73	10	−7.29	48.57	78.38	0.58 (0.45 – 0.72)
(b) BN	73	10	−1.43	85.71	63.16	0.79 (0.69 – 0.89)****
**(3) Words in….**						
(a) BN	45	20	−0.83	92.0	85.0	0.91 (0.82 – 0.99)****

^Cutoff calculated as Youden’s Index SWN, speech-weighted noise; BN, babble noise; SNR, signal-to-noise ratio; dB, decibels; ROC AUC, area under the receiver operating characteristic curve; CI, Confidence Intervals (95%). *p < 0.05; ****p < 0.0001. p-values test the null hypothesis that the AUC = 0.5. p values in bold represent significant differences between groups (p < 0.05).

### Classification Value of Speech Discrimination Tasks to Classify All Mild (Early and Late) pwMS From Controls

A logistic regression model was developed using speech discrimination abilities to classify those without MS (controls = 0), and those with mild MS (coded as 1). MS participants in the logistic regression had a median EDSS score of 0, thereby making this group indistinguishable from a healthy population based on functional scores alone. The purpose of the logistic regression was not for diagnostic value, but to determine how SiN tasks differentiated between subjects with the presence of subclinical MS pathology and controls, and therefore, how well it could monitor or identify changes during insidious disease course.

To be clinically viable, assessments should be quick and easy to administer, therefore, part of the model building strategy was to only consider speech discriminated at certain SNRs as predictor variables (2–3 min testing at each SNR). Midpoint curve SNRs require the whole psychometric function to be obtained; taking anywhere between 10 and 20 min to administer the test (refer to Table 6); compromising tolerability of the test. Based on the two-way ANOVAs described in Figures 3–5, performances at specific SNRs in all three BN tasks (sentences, keywords, and words) were considered. A maximum of two fixed effects were used in any model to avoid overfitting the data with a small data set (45 observations). Ten possible regression models (Supplementary Table 1A) were generated using MATLAB Statistic Toolbox Release 2019b and based on the SiN performances at SNRs of 1 and −1 dB only as these SNRs were closest to the midpoint of the psychometric functions previously detailed, and therefore the steepest points for classification. A maximum of two fixed effects were used in any model to avoid overfitting the data with a small data set (45 observations). The final model, based on the lowest AIC, had two fixed effects: word discrimination in BN (total/30) and sentence discrimination in BN (total/10) at an SNR of −1 dB (model 9 in bold in Supplementary Table 1A). Variables had a variance inflation factor (VIF) of 1.20. This was well below the recommended cut off VIF of 5, indicating no problematic levels of multicollinearity among predictors.

Table 7 presents the results from a log likelihood ratio test to ascertain if the model with the SiN predictors was more effective than a null model (intercept only). The results of the test suggest that the null model should be rejected in favor of the logistic regression model using SiN measures as predictors χ^2^(2) = 19.7, *p* < 0.0001.

**TABLE 7:** Logistic regression model used to classify controls (*n* = 20) and all mild MS (*n* = 25).

						95% C.I for e^β^	
Predictor	Estimate (β)	*SE* β	tStat	*p*	OR (e^β^)	Lower	Upper
Intercept	7.88	3.06	2.57	0.01			
Sentences presented in babble at SNR of −1 dB	−1.24	0.46	−2.72	0.007	0.29	0.12	0.73
Words presented in babble at SNR of −1 dB	−0.40	0.18	−2.22	0.027	0.67	0.47	0.96

		**χ^2^**	***df***	***p***			

Overall model evaluation							
*Likelihood ratio test*		19.7	2	<0.0001			

*SNR, signal-to-noise ratio; SE, standard error; OR, odds ratio; C.I, confidence interval. MATLAB 2019b statistical package was used. Ordinary R^2^ = 0.35. Adjusted R^2^ = 0.32. 45 observations, 42 error degrees of freedom.*

The parameter estimates of fixed effects are also listed in Table 7, along with the standard error, t statistic, *p*-values, odds ratio (OR) and CIs (95%). Statistical significance of individual regression coefficients (βs) were tested using the *t*-statistic (testing the null hypothesis that β is equal to zero). Total sentences and words correctly discriminated in BN at an SNR of −1 dB were significant discriminators of controls from MS participants (*p* < 0.05). For a one unit increase in sentence discrimination performance (i.e., an additional sentence correctly recalled), when all other variables in the model are held constant, the expected OR of the participant being a pwMS was 0.29 (95% CI: 0.12 –0.73); i.e., 71% (1-e^–1.24^) reduced odds of the participant being a pwMS. There was also an effect of word discrimination, where, for a one unit increase in word discrimination performance, the expected OR of the participant being a pwMS was 0.67 (95% CI: 0.47 –0.96); i.e., 33% (1-e^–0.40^) reduced odds of the participant being a pwMS.

**FIGURE 6:**
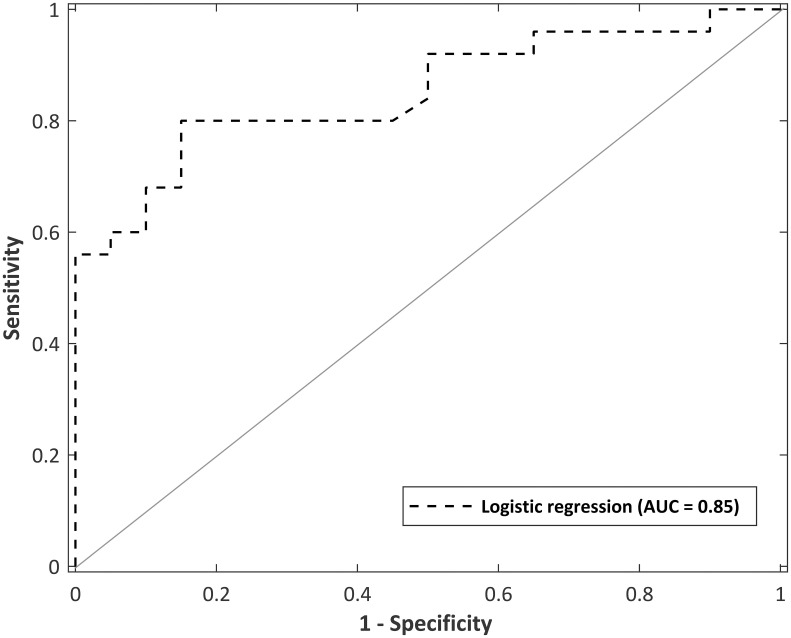
Receiver operating curve for a logistic regression model with predictor variables of total sentences and words at an SNR of –1 dB in multi-talker babble (total administration time of 5 min). Area under the curve (AUC) = 0.85.

The logistic regression model was evaluated using fivefold cross validation, split into a 70:30 training/test set. A receiver operating characteristic (ROC) curve is visualized in Figure 6 to evaluate the logistic regression as a discrimination tool. The AUC-ROC was 0.85, considered to be excellent classifying performance (Hosmer, 2000). The cutoff point (Youden’s J statistic) was 0.59, and this was used to classify participants into controls and MS. The predicted vs. observed classifications are presented as a confusion matrix in Table 8. The model has 80% sensitivity and 85% specificity in the classification of participants. The classification model is not suggested to be used as a diagnostic tool, but as a way to distinguish controls with normal neurological functioning from people with mild MS with subtle neurological dysfunction that goes undetected by the EDSS. Given that the logistic regression has 80% sensitivity in detecting subtle MS deficits, the confusion matrix provides evidence for the 5-min SiN predictors to be a useful clinical tool.

**TABLE 8:** The observed and the predicted classifications between controls and all mild MS using a logistic regression based on a 5-min speech-in noise task by the cutoff of 0.59.

	Predicted		
Observed	Control	All mild MS	% Correct
**Control**	17	3	85.0%.
**All mild MS**	5	20	80.0%
**Overall % correct**			82.22%

*Sensitivity = 20/(20 + 5)% = 80%. Specificity = 17/(17 + 3)% = 85%. False positive = 3/(3 + 17)% = 15%. False negative = 5/(5 + 20)% = 20%.*

## Discussion

Quantifying functional deficits in early MS remains a considerable challenge in the field (Cree et al., 2019); yet it is paramount for tailoring disease-modifying strategies and evaluating potential therapeutic candidates. This study evaluated the sensitivity of SiN tasks to measure early sensory and cognitive changes in pwMS. Three main results emerged from the current study: (1) SiN tasks, particularly those with a multi-talker babble background, sensitively detected speech discrimination deficits in early and mild pwMS (median EDSS = 0) with normal hearing; (2) there were mild/moderate correlations between SiN metrics and standardized neuropsychological assessments which indicate that pwMS with lower functional scores also had poorer speech discrimination in multi-talker babble; (3) a quick 5-min task with words and keywords presented in multi-talker babble at a single SNR was 82% accurate in classifying mild pwMS (median EDSS = 0) from healthy controls. Together, this indicates that SiN tasks measure MS-disturbances on a scale order of magnitude more sensitively (≤20% reduction in speech intelligibility in babble for pwMS compared to controls) than standard EDSS steps at the early and mild stages of the disease.

### Multi-Talker Babble: A Potent Masker for Mildly Affected MS

Speech-in-noise deficits depended on noise type: speech-weighted noise measured zero to modest MS impairments in discrimination, whilst multi-talker babble elicited significant MS impairments at almost all SNRs (except for floor and ceiling SNRs) for all linguistic stimuli. Speech-weighted noise is an energetic masker that diminishes target audibility only through masking and blending of acoustic signals at the periphery (Bronkhorst, 2000). In contrast, multi-talker babble elicits confusion because of its similarity to speech and its saliency which will involuntarily capture attention (Bronkhorst, 2000). Our findings that SiN performance was disrupted only in multi-talker babble and not in speech-weighted noise shows that the SiN difficulties in people with mild MS are not due to linguistic difficulties but must be due to cognitive disruption. Speech degradation in babble in pwMS might also be related to impaired temporal-resolving capacity. Both Mustillo (1984) and Rappaport et al. (1994) postulated that a contributing factor to SiN deficits in pwMS could be a deficit in temporal processing, possibly related to the delay of signal transmission within the auditory pathways. Complex acoustic signals, such as natural masking speech babble, have temporally fluctuating levels, where listeners can use a ‘glimpsing’ strategy (Li and Loizou, 2007) to extract information in “a time-frequency region which contains a reasonably undistorted ‘view’ of local signal properties” (Simpson and Cooke, 2005). Such fluctuations are not present in the steady state speech-weighted noise, therefore, the temporal resolution skills required to use glimpsing to distinguish speech from babble may partly explain why it is such a potent masker for early MS. This is consistent with the findings of Rappaport et al. (1994) that pwMS were significantly impaired in discriminating monosyllables presented monaurally against interrupted noise at every SNR, but not in continuous noise. It was concluded that pwMS, particularly those with forebrain lesions, had impoverished temporal processing deficits, as they could not discriminate speech fragments in the silent periods. When a target signal is degraded due to reduced auditory temporal abilities or attentionally demanding characteristics of multi-talker babble, MS listeners must place greater demands on finite cognitive processes to reconcile perceptual ambiguity– a demand that is further exacerbated in smaller or negative SNRs.

### Lack of Auditory Complaints in Everyday Life

Although speech discrimination in babble was impaired in early and late mild pwMS groups, we did not identify any subjective difficulties in daily life by pwMS (see Table 1) using the self-report Auditory Attention and Distress Questionnaire (AADQ). The AADQ was developed by our group, and has identified changes in everyday life in auditory tasks in high-performing ASD people (Dunlop et al., 2016) and in advanced stages of MS (Iva et al., 2021). We propose that deficits in SiN tasks in babble reflect a cognitive deficit that has not yet impacted auditory performance and processing in daily life settings in pwMS. The absence of complaints might reflect redundant auditory processing (Furst and Levine, 2015), either intrinsic [multiple parallel auditory CNS representations (Musiek, 1986)] or extrinsic [syntactic and semantic cues, or multimodal information through (say) lipreading] (Wu et al., 2015). Further, early pwMS may use neural compensatory mechanisms to reduce or mask functional deficits (Audoin et al., 2005).

### SiN Performance Does Not Reflect Disease Duration

Both early and late mild MS groups were significantly impaired on all SiN tasks presented in multi-talker babble compared to healthy controls, but no differences were revealed between the MS groups. Both physically (EDSS) and neuropsychologically (cognitive tests used here), the two groups were functionally very similar, despite the significant difference in disease duration. There is contradictory evidence regarding disease duration on cognitive profiles (Brochet and Ruet, 2019) and on the relationship between cognitive impairment and level of physical disability (Lynch et al., 2005). Regardless, the functional preservation in the late MS group as measured by standardized clinical measures, is supported by the fact that early and late discrimination abilities within our SiN tasks were also very similar. The only task that differentiated early and late mild pwMS was the LDL; a sensory test typically used to evaluate hypersensitivity to sound (Sheldrake et al., 2015). Overall, late mild pwMS reported significantly more discomfort than controls and early mild pwMS to multi-talker and speech stimuli presented at various intensities (dB), suggesting that despite similar discrimination performances and subjective experiences in daily life, late mild pwMS had less tolerance to louder stimuli than early pwMS. This could have implications for social avoidance and fatigue in sustained exposure to such acoustic environments.

### SiN Measures Correlate With Standard Neuropsychological Tasks

PwMS with cognitive deficits are likely to struggle with SiN tasks, particularly when temporal processing deficits further degrade the signal and thereby exacerbate demand on top–down processes. Interpreting SiN discrimination deficits as a reflection of cognitive MS impairments is supported by our finding of significant negative correlations between SNRs at 50% discrimination accuracy and standardized neuropsychological performance in the PASAT and CVLT. The PASAT is a complex test of mental arithmetic, attention, working memory, information processing speed and places a heavy load on executive control processes (Gronwall, 1977). The CVLT-II is a measure of episodic verbal learning and memory (Delis et al., 1987); a test which is particularly sensitive in early MS as verbal memory deficits have been reported in pwMS with a mean duration of 1.5 years (Amato et al., 1995). The digit span test (DST- WAIS-IV) did not correlate with any of our SiN tests. This may seem surprising given that the DST is referenced as a standardized measure of memory; however, the demands on short-term memory capacity in the digit span test are likely to be minimal. The digit span test requires participants to repeat a series of digits of increasing length that are orally presented at a one digit per second rate in silence. Normal forward digit spans (seven ± two digits) have been reported in amnestic patients with Alzheimer’s disease, Korsakoff’s syndrome (Cullum, 1998), and early phase MS (<4 years since diagnosis) (Landrø et al., 2000). Backwards Digit span requires different processes or strategies as the task demands require mentally reversing the perceived sequence (St Clair-Thompson and Allen, 2013), but are also normal in early MS (Landrø et al., 2000). None of the discrimination tasks in speech-weighted noise correlated with any neuropsychological task, consistent with the idea that higher phonological mismatches in babble results in more effortful processing mechanisms based on working memory to make speech comprehensible. However, we must point out that a limitation of our interpretations is that only pwMS were neuropsychologically tested. The cognitive functions required for speech discrimination in noise should ideally be examined in controls as well.

### The Utility of SiN Tasks

Words, compared to sentences, elicited a greater degree of discrimination impairment in MS listeners. It may be that mild pwMS can exploit contextual and semantic cues present in sentences that are absent when words are presented in isolation. Reading comprehension and general linguistic competence aids SiN intelligibility (Avivi-Reich et al., 2014; Heinrich and Knight, 2016), however the relationship is described in tests utilizing sentences- but not syllables (Heinrich and Knight, 2016). Words in isolation are likely to provide relatively more ‘bottom–up’ acoustic-phonetic cues, in contrast to sentences (Pisoni, 1996). The role of linguistic cues in more difficult and longer sentences could be investigated in future studies. We note the efficacy of our logistic regression model in classifying minimally impaired pwMS from healthy controls using predictors of sentence and word discrimination in babble at an SNR of −1 dB. MS participants included in the logistic regression had a median EDSS score of 0; making this group indistinguishable from a healthy population based on EDSS scores alone. In contrast to this, SiN measures have high sensitivity and specificity for the presence of MS pathology – which suggests that it may be a viable approach to detecting and monitoring MS-related neuropsychological changes not covered by the EDSS. Supporting this, correlations with EDSS scores and neuropsychological impairment or brain changes measured by MRI are weak or non-existent (Cohen et al., 1993). Currently, progression of disease measured by EDSS is the most important primary and secondary endpoint in MS trials addressing the efficacy of clinical interventions (Meyer-Moock et al., 2014). It may be advantageous to use SiN measurements in conjunction with the EDSS to provide information on such sensory and cognitive changes that aren’t well addressed by the EDSS. Longitudinal studies will need to be carried out to confirm its clinical utility in monitoring disease, however, this is the first preliminary step in establishing the viability of SiN tests in the clinic.

### Hidden Hearing Loss

Difficulty hearing conversational speech in listeners with clinically normal thresholds has received more attention recently (Carney, 2018), and is therefore an aspect that should be considered in the interpretation of results. Hidden hearing loss (HHL) is believed to be damage biased toward low spontaneous firing rate neurons which are critical for coding suprathreshold sounds (Grinn et al., 2017). There are no general standard tests of HHL but that commonly (e.g., Bernstein and Trahiotis, 2016) this is tested for by using sounds presented at levels close to those experienced in normal conversations. Two suprathreshold evaluations were incorporated in our test battery – firstly, we examined loud discomfort levels (LDLs) and found that there were no differences between control and MS participants except for the late mild MS listeners for noise and for high levels of speech. Secondly the questionnaire, which examines the lived experience of participants in common daily life situations (cafes, supermarkets, halls, etc.), revealed no abnormalities in either the early or late mild MS groups – despite significant deficits in SiN tasks. Despite this, both the early and late mild MS groups still did significantly worse in the SiN tasks that employed babble as a background masker. Both these factors make it very unlikely that HHL was responsible for the effects observed in the SiN tasks.

## Conclusion

The capacity for SiN tasks to measure subtle deficits specific to early MS may reflect their dependence upon dynamic heterarchical interactions between central auditory processing and cognition. Acoustic analysis of complex auditory scenes entails both exquisite local neural timing and the integrity of diffuse, higher-level networks, revealing subtle changes in MS that are not reflected in the EDSS. We do not suggest that our SiN task might replace more conventional neuropsychological measures in MS but suggest that it might be employed as an additional measure for changes in cognitive function, due to its ease of use and speed. Our SiN task only takes 5 min to administer, and it is cost effective and non-invasive, these features are advantageous in a clinical setting.

## Data Availability Statement

The original contributions presented in the study are included in the article/Supplementary Materials, further inquiries can be directed to the corresponding author/s.

## Ethics Statement

The studies involving human participants were reviewed and approved by the Monash University Human Research Ethics Committee. The patients/participants provided their written informed consent to participate in this study.

## Author Contributions

PI conducted the experiments, analyzed the data, and co-wrote the manuscript. JF, MC, OW, and BG aided in data collection and review of the manuscript. RM aided in data analysis and review of the manuscript. RR conceptualized and designed the experiments, and co-wrote the manuscript.

## Conflict of Interest

The authors declare that the research was conducted in the absence of any commercial or financial relationships that could be construed as a potential conflict of interest.
