# Supplementary material for: Speech Discrimination Tasks: A Sensitive Sensory and Cognitive Measure in Early and Mild Multiple Sclerosis
Source: Front Neurosci. 2020 Dec 23;14:604991. doi: 10.3389/fnins.2020.604991 (PMC7786116; doi:10.3389/fnins.2020.604991)
Supplement: Supplementary file 1 [file Table_1.DOCX]

Supplementary Material

# Supplementary Data

**Auditory Attention and Difficulty Questionnaire (AADQ)**

**Item number and statement**

1. I find the sound of doorbells annoyingly loud

2. I find the sound of a telephone ringing to be uncomfortably loud

3. I find restaurants and cafes to be uncomfortably loud.

4. I have arguments with my family or friends because I think they talk too loudly.

5. I have the TV or radio volume much lower than do my family or friends

6. I find supermarkets to be uncomfortably loud

7. The sounds of running water, like a toilet or shower, are uncomfortably loud

8. I can hear the sounds of birds singing in the mornings

9. The sounds of building work are painfully loud

10. Traffic noises are uncomfortably loud

11. The sound of screeching tyres is uncomfortably loud.

12. When I am in a theatre watching a movie or play, I find it uncomfortably loud when people around me are whispering and rustling packets

13. I have trouble understanding others when an air conditioner or fan is on

14. Unexpected sounds, like a smoke detector or alarm bell, are uncomfortable

15. I avoid social gatherings (like parties) because I find the noise levels annoying

16. I find parties are too loud to be able to concentrate to have a conversation

17. I can understand conversations even when several people are talking

18. I have difficulty hearing a conversation when I'm with one of my family at home

19. I have difficulty following a conversation on the phone or mobile when I'm at home

20. When I am having a quiet conversation with a friend, I have difficulty understanding them

21. When I'm seeing my doctor in his/her rooms, it is hard to follow the conversation

22. I have trouble understanding dialogue in a movie or at the theatre

23. When I am talking with someone across a large empty room, I have difficulty understanding what they say

24. I miss a lot of information when I'm listening to a lecture or a public talk

25. When a speaker is addressing a small group, and everyone is listening quietly, I have to strain to hear

26. I have to ask people to repeat themselves in one-on-one conversations in a quiet room

27. I have trouble understanding a waiter/waitress in a quiet restaurant

28. When I am in a small office, talking or answering questions, I have difficulty following the conversation

29. When I am having dinner with several other people, I have difficulty following the conversation because I find it hard to identify who is speaking

30. I have difficulty communicating with others when we are in a crowd

31. When I am in a crowded supermarket talking with the cashier, I can follow the conversation*

32. I have difficulty understanding a shop assistant in a crowded shop

33. In social situations I often feel left out because people think I have difficulty following the conversations

**Supplementary Figure A.1.** The 33 items on the Auditory Attention and Difficulty Questionnaire (AADQ) were Modified from the University of Auckland Evaluation of Hearing Performance, the Amsterdam Inventory, The Denver Scales (Schow & Nerbonne JARA 1980), and the Hearing Handicap Inventory for Adults – Screening (Ventry, I. & Weinstein, B. Ear Hear 1982). Statements were summarized into three components: Component 1, the Audio-Attentional Difficulty subscale, measured difficulties attending to speech in noisy environments from fourteen items (items 18-30, 33). Component 2, the Auditory Discomfort (Non-Verbal) subscale, measured discomfort to non-verbal environmental sounds from eight items (items 1, 2, 4, 9-12, 14). Component 3, the Auditory Discomfort (Verbal) subscale, measured discomfort to verbal sounds from seven items (items 3, 6, 13, 15, 16, 31, 32). The questionnaire was completed on paper during the testing session under no time restriction.

* Item 31 had negative valence and so participants’ responses were reversed.


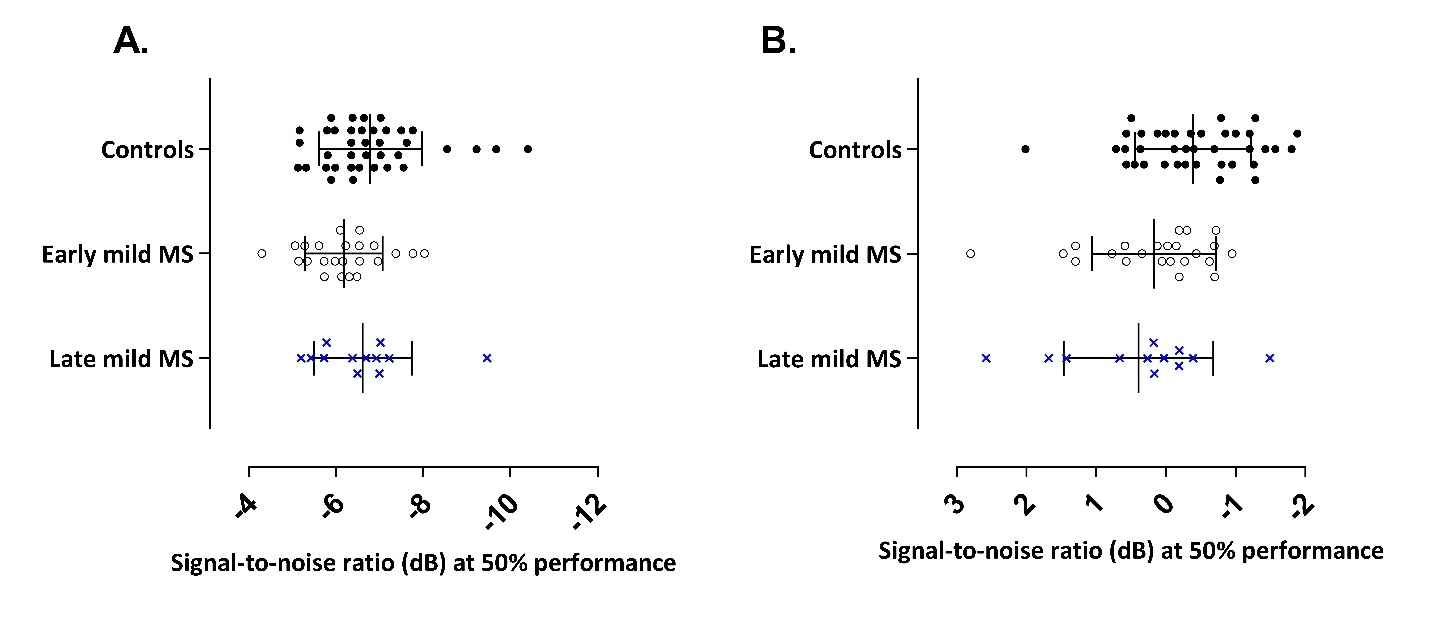


**Supplementary Figure A.2. Scatter plots presenting the signal-to-noise ratios at 50% sentence discrimination in speech-weighted noise (A) and multi-talker babble (B) for individual participants.** Lines represent the mean SNRs ± SD (dB) for controls (n = 38, filled circles), early (n = 23, open circle) and late mild MS subjects (n = 12, cross). SNRs were calculated from the midpoints of Boltzmann sigmoidal functions fitted to each participant.


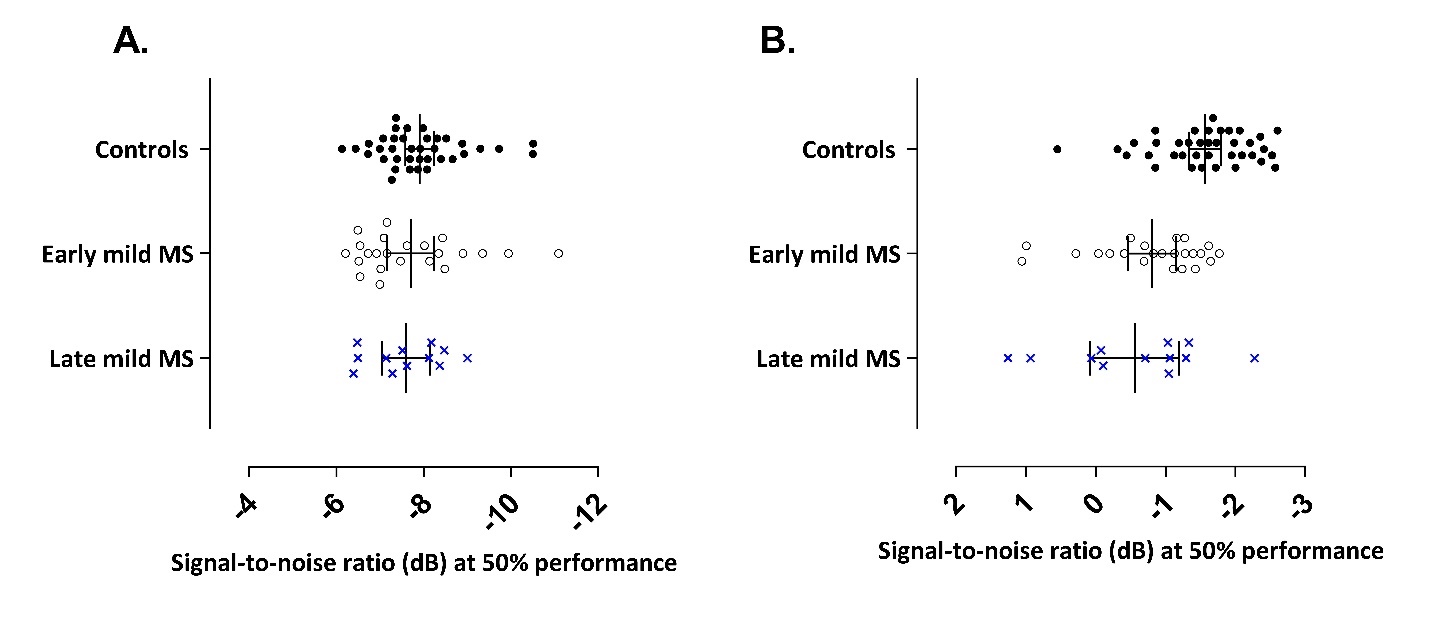


**Supplementary Figure A.3. Scatter plots presenting the signal-to-noise ratios at 50% keyword discrimination in speech-weighted noise (A) and multi-talker babble (B) for individual participants.** Lines represent the mean SNRs ± SD (dB) for controls (n = 38, filled circles), early (n = 23, open circle) and late mild MS subjects (n = 12, cross). SNRs were calculated from the midpoints of Boltzmann sigmoidal functions fitted to each participant.


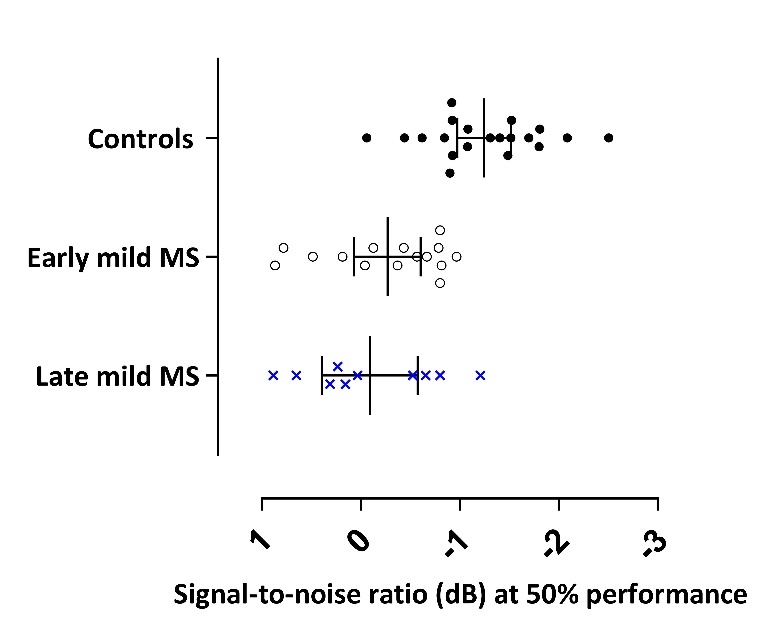


**Supplementary Figure A.4. Scatter plots presenting the signal-to-noise ratios at 50% word discrimination in multi-talker babble for individual participants.** Lines represent the mean SNRs ± SD (dB) for controls (n = 38, filled circles), early (n = 23, open circle) and late mild MS subjects (n = 12, cross). SNRs were calculated from the midpoints of Boltzmann sigmoidal functions fitted to each participant.

| **Supplementary Table 1A. AIC comparisons of logistics regression models used to classify controls from mild pwMS** | |
| --- | --- |
| **Variables in model (with intercept)** | **Akaike’s Information Criterion (AIC)** |
| **Variables at SNR of 1dB:** |  |
| (1) Words at SNR 1dB | 53.69 |
| (2) Keywords at SNR 1dB | 62.76 |
| (3) Sentences at SNR 1dB | 65.81 |
| (4) Words at SNR 1dB and Keywords at SNR 1 dB | 54.89 |
| (5) Words at SNR 1dB and Sentences at SNR 1 dB | 55.34 |
| **Variables at SNR of -1dB:** |  |
| (6) Words at SNR -1dB | 56.82 |
| (7) Keywords at SNR -1dB | 54.07 |
| (8) Sentences at SNR -1dB | 59.69 |
| **(9) Words at SNR -1dB and Keywords at SNR -1 dB** | **51.31** |
| (10) Words at SNR -1dB and Sentences at SNR -1 dB | 55.73 |
| AIC = Akaike’s Information Criterion score is a comparative value that evaluates goodness-of-fit between models. The model with the lowest AIC indicates a superior balance between goodness of fit and avoiding overfitting the data | |
